# Supplementary material for: Epigenetic and Epitranscriptomic Antiviral Responses in Plants for Disease Management
Source: Viruses. 2025 Dec 22;18(1):17. doi: 10.3390/v18010017 (PMC12846519; doi:10.3390/v18010017)
Supplement: Supplementary file 1 [file viruses-18-00017-s001.zip › viruses-3881732-supplementary.pdf]

Supplementary Table 1. Estimated annual economic losses in major crops due to plant pathogenic viruses worldwide. Values are approximate and compiled from published articles.

**Region/Country.**	**Crop (s)**	**Estimated Loss (USD, approx.)**	Causative Virus	**Reference (s)**
Africa/South Asia	Cassava	1.9-2.7 billion	Cassava mosaic begomoviruses	Tatineni & Hein, 2023
USA	Potato	100-120 million	Potato leafroll Polerovirus	Sastry & Zitter, 2014; Wale et al., 2008
United Kingdom (UK)	Cereals (Barley,Oats,Rice,Wheat,Maize)	10-15 million	*Barley yellow dwarf virus*	Ordon et al., 2009
South-East Asia	Rice	~1.0 billion	Rice tungro viruses	Abo & Sy, 1997; Hull & Centre, 2014
USA, Australia, Eastern Europe	Tomato, Lettuce, Eggplant, Pepper	1.0-1.5 billion	*Tomato spotted wilt virus*	Tatineni & Hein, 2023
Bangladesh	Potato	0.5-1.8 billion	Potato leafroll virus, Potato virus X, Potato virus Y, Potato virus S, Potato virus H, Potato aucuba mosaic virus and Potato virus M	Rashid et al., 2020
